# Insights into deregulated TNF and IL-10 production in malaria: implications for understanding severe malarial anaemia

**DOI:** 10.1186/1475-2875-11-253

**Published:** 2012-08-01

**Authors:** Philippe S Boeuf, Séverine Loizon, Gordon A Awandare, John KA Tetteh, Michael M Addae, George O Adjei, Bamenla Goka, Jørgen AL Kurtzhals, Odile Puijalon, Lars Hviid, Bartholomew D Akanmori, Charlotte Behr

**Affiliations:** 1 Institut Pasteur, Unité d’Immunologie Moléculaire des Parasites URA CNRS 2581, Paris, France; 2 Immunology Department, Noguchi Memorial Institute for Medical Research, College of Health Sciences, University of Ghana, Accra, Ghana; 3 Department of Hematology, School of Allied Health Sciences, College of Health Sciences, University of Ghana, Accra, Ghana; 4 Department of Child Health, University of Ghana Medical School, College of Health Sciences, University of Ghana, Legon, Accra, Ghana; 5 Centre for Medical Parasitology, Copenhagen University Hospital (Rigshospitalet), Copenhagen, Denmark; 6 Department of Clinical Microbiology, Copenhagen University Hospital (Rigshospitalet), Copenhagen, Denmark; 7 Department of Infectious Diseases, Copenhagen University Hospital (Rigshospitalet), Copenhagen, Denmark; 8 Department of International Health, Immunology, and Microbiology (ISIM), University of Copenhagen, Copenhagen, Denmark; 9 UMR CNRS 5164, Bordeaux, France; 10 Université de Bordeaux, Bordeaux, France

**Keywords:** Malaria, Anaemia, Cerebral malaria, Severe malarial anaemia, Monocytes, T cells, CD69, HLA-DR, Monocyte de-activation, TNF, IL-10, Cytokines

## Abstract

**Background:**

Severe malarial anaemia (SMA) is a major life-threatening complication of paediatric malaria. Protracted production of pro-inflammatory cytokines promoting erythrophagocytosis and depressing erythropoiesis is thought to play an important role in SMA, which is characterized by a high TNF/IL-10 ratio. Whether this TNF/IL-10 imbalance results from an intrinsic incapacity of SMA patients to produce IL-10 or from an IL-10 unresponsiveness to infection is unknown. Monocytes and T cells are recognized as the main sources of TNF and IL-10 *in vivo*, but little is known about the activation status of those cells in SMA patients.

**Methods:**

The IL-10 and TNF production capacity and the activation phenotype of monocytes and T cells were compared in samples collected from 332 Ghanaian children with non-overlapping SMA (n = 108), cerebral malaria (CM) (n = 144) or uncomplicated malaria (UM) (n = 80) syndromes. Activation status of monocytes and T cells was ascertained by measuring HLA-DR^+^ and/or CD69^+^ surface expression by flow cytometry. The TNF and IL-10 production was assessed in a whole-blood assay after or not stimulation with lipopolysaccharide (LPS) or phytohaemaglutinin (PHA) used as surrogate of unspecific monocyte and T cell stimulant. The number of circulating pigmented monocytes was also determined.

**Results:**

Monocytes and T cells from SMA and CM patients showed similar activation profiles with a comparable decreased HLA-DR expression on monocytes and increased frequency of CD69^+^ and HLA-DR^+^ T cells. In contrast, the acute-phase IL-10 production was markedly decreased in SMA compared to CM (*P* = .003) and UM (*P* = .004). Although in SMA the IL-10 response to LPS-stimulation was larger in amplitude than in CM (*P* = .0082), the absolute levels of IL-10 reached were lower (*P* = .013). Both the amplitude and levels of TNF produced in response to LPS-stimulation were larger in SMA than CM (*P* = .019). In response to PHA-stimulation, absolute levels of IL-10 produced in SMA were lower than in CM (*P* = .005) contrasting with TNF levels, which were higher (*P* = .001).

**Conclusions:**

These data reveal that SMA patients have the potential to mount efficient IL-10 responses and that the TNF/IL-10 imbalance may reflect a specific monocyte and T cell programming/polarization pattern in response to infection.

## Background

In sub-Saharan Africa, severe malarial anaemia (SMA) is a frequent complication of *Plasmodium falciparum* infections in young children [1] and is one of the main causes of severe anaemia, with a case-fatality rate reaching 23% in malaria holoendemic areas [2]. Pathogenesis of SMA is not well understood, although destruction of the infected erythrocytes accompanied by clearance of uninfected erythrocytes, erythropoietic suppression and dyserythropoiesis, can all contribute to anaemia [3,4]. SMA is associated with elevated levels of myelo-suppressive cytokines, such as TNF, but this is not specific to the SMA syndrome, as children with cerebral malaria (CM) also have highly elevated TNF plasma levels [5]. Previous analysis showed that SMA can be distinguished from CM on the basis of an elevated ratio of TNF to its potent anti-inflammatory regulator IL-10 suggesting a central role for the TNF-IL-10 balance in SMA pathogenesis [5,6]. This is supported by the observation that anaemia is increased in IL-10 knockout mice infected with *Plasmodium chabaudi*[7] and reversed upon TNF neutralization [7,8].

TNF alone and in concert with multiple other cytokines and chemokines is a potent inhibitor of haematopoietic stem cells [9]. Elevated levels of TNF in patients with chronic inflammation [10], aplastic anaemia [11] or inherited anaemic disorders [12] have been associated with inhibition of erythropoiesis. Multiple underlying mechanisms have been reported, including the caspase-mediated cleavage of the major erythroid transcription factor GATA-1 [13], impairment of cell cycle progression [14], and remodelling of the extracellular matrix within erythroid niches [15]. The high TNF/IL-10 ratio characteristic of SMA patients might reflect an insufficient production of IL-10 in SMA patients to prevent or counteract the inhibition of erythropoiesis and the increase of erythrophagocytosis induced by TNF and/or to mitigate other pro-inflammatory stimuli.

Monocytes and T cells are generally recognized as the main source of TNF and IL-10 *in vivo*. However, little is known about their activation status and their contribution to the TNF/IL-10 imbalance associated with SMA. Data on monocyte or T cell status in SMA patients are scarce, although indirect lines of evidence suggest activation of the monocyte/macrophage compartment in SMA patients [16-19]. To date, no published study has compared T cell and monocyte status in SMA and CM.

The work reported here sought at investigating whether children with SMA or CM have distinct TNF and IL-10 production capacities accounting for their different TNF/IL-10 ratios. To gain insight into the cytokine production capacity and the cellular subsets involved, Ghanaian children with non-overlapping SMA or CM syndromes were recruited. Their T cell and monocyte activation statuses were compared. In addition, their intrinsic TNF and IL-10 secretion capacity was investigated using whole-blood stimulation assays with LPS and PHA, used as surrogate of monocyte and T cell stimulant, respectively. Similar analyses were performed on children with uncomplicated malaria (UM), considered as a control group for acute malaria and asymptomatic children (AC) living in the same area, recruited as a reference group for population baseline. The data show that the low IL-10 level in SMA cannot be attributed to a defective IL-10 response capacity and point to a specific dysregulation. Moreover, the parameters investigated provide interesting novel insights into the distinct inflammatory statuses in SMA and CM.

## Methods

### Study site, patient recruitment and sample collection

Venous blood was collected in sterile heparinised tubes from children (aged 1–12 years) enrolled at the Department of Child Health, Korle-Bu Teaching Hospital, Accra, during the peak malaria transmission season (July to August) in 2001 and 2003. Signed informed consent was obtained from the parents or guardians. The ethics and protocol review committee of the University of Ghana Medical School approved the study. Criteria for patient enrolment and classification have been detailed elsewhere [20]. Briefly, patients with asexual *P*. *falciparum* parasitaemia ≥ 5,000 parasites/μL of blood and axillary temperature > 37.5°C were further categorized into UM, SMA and CM. UM was defined as full consciousness, haemoglobin (Hb) ≥ 8 g/dL, and no other complications of malaria. SMA patients had Hb levels < 5 g/dL, with full consciousness and no other known cause of anaemia. CM was defined as unrousable coma with a Blantyre coma score ≤ 3 for more than an hour and without any sign of other possible causes of coma and Hb levels ≥ 5 g/dL. Children with a positive sickling test or any clinical presentation other than malaria were excluded from the study. Convulsing patients were excluded from the SMA and UM groups. Patients included in the study were followed up and blood samples were taken at 3 and 7 days post admission. Asymptomatic healthy children (AC) were recruited in Dodowa, a community nearby Accra, from a random sample of an existing cohort of pre-school and school children and matched for age and sex with the patients. Hb concentration and complete blood cell count were determined using an automated haematology analyzer (Sysmex). Parasitaemia was determined from Giemsa-stained thick and thin blood films and expressed as parasitized red cells per μL of blood, based on individual white blood cell counts as described [21]. As the patients recruited in 2001 and 2003 showed similar clinical, haematological and parasitical characteristics, the data were pooled.

### Flow cytometry

Monocytes and T cells were identified on the basis of their forward/side scatter profiles and expression of CD14 (RM052, Immunotech) and CD3 (SK7, Becton Dickinson) respectively. Monocyte activation status was assessed by HLA-DR (L243, Becton Dickinson) surface expression as described [22]. CD69 (L78, Becton Dickinson) and HLA-DR expression was used to determine T cell activation [23]. Staining was conducted on whole blood using saturating amounts of fluorochrome-conjugated specific or control antibodies (Becton Dickinson) for 30 minutes at room temperature. Red blood cells were lysed, and white blood cells washed, fixed in 0.5% formaldehyde before acquisition within the next 24 hour on a FACScan flow cytometer (Becton Dickinson). A minimum of 5,000 positively-stained events were acquired. Staining was analysed using CellQuest 3.3 software (Becton Dickinson).

### Whole-blood stimulation

Within four hours after venous blood collection, cytokine production was assessed following a 24-hour incubation of 500 μL of heparinised whole blood with or without addition of 10 μl of lipopolysaccharide (LPS, *E*. *coli* O111:B4; Sigma-Aldrich) or phytohaemagglutinin (PHA; Sigma-Aldrich) diluted in RPMI 1640 (Life technologies, Invitrogen) such that the final concentration was 100 ng/mL and 2 μg/mL for LPS and PHA respectively. Assays were conducted in polypropylene tubes in the presence of penicillin and streptomycin at 37°C in 5% CO_2_. At the end of the incubation period, the samples were centrifuged and supernatants stored at −80°C until cytokine assays. IL-10 and TNF levels released in the supernatant of un-stimulated whole-blood culture after 24-hour, reflect plasma concentrations at admission [21] and is referred as the “spontaneous cytokine secretion”.

### Cytokine assays

Concentrations of IL-10 and TNF in supernatant from whole blood assays were quantified in duplicate using commercially available ELISA kits (BioSource) according to the manufacturer’s recommendations. Cytokine production capacity was calculated as the cytokine concentration measured in the supernatant of LPS or PHA stimulated whole blood divided by the concentration of cytokines produced in the un-stimulated condition i.e. the “spontaneous cytokine production” and reported as the fold-increase from the un-stimulated condition.

### Assessment of pigmented monocyte density

Malaria pigment-containing monocyte densities were determined by counting 30 monocytes on thin films by an experienced haematologist blinded to clinical presentation and outcome, as described elsewhere [24].

### Statistical analyses

Pearson-Chi-square test was used to compare qualitative data across groups. Quantitative data were compared across three or more groups by Kruskal-Wallis test, while the Mann–Whitney test was used for pairwise comparisons. *P* values less than .05 were considered significant. Associations between different variables were analysed by Spearman’s rank correlation and considered statistically significant if r > 0.25 and *P* < .05.

## Results

### Clinical, parasitological and haematological characteristics of malaria patients

The clinical, parasitological and haematological characteristics of the three groups of malaria patients at admission are shown in Table 1. Age, gender, parasitaemia, leukocyte and monocyte counts did not differ between groups. Lymphocyte counts were not significantly different in SMA and CM patients and both severe groups had higher counts than UM patients (*P* ≤ .0051). As expected, erythrocyte counts were lower in SMA than in CM or UM (*P* ≤ .0001). The percentage of circulating pigmented monocytes differed between the three groups, being lower in SMA than in CM (*P* = .032), but higher than in UM (*P* = .039) (Table 1).

**Table 1:** Clinical and biological parameters of Patients characteristics

	**SMA**	**CM**	**UM**	**P value**	**P value**
				**SMA vs. CM (1)**	**SMA vs. CM vs. UM (2)**
No. children	108	144	80		
Age (years)	3.5 (1–5)	3.1 (1–6)	3.8 (2–6)	NS^b^	NS^b^
Sex [*n* (%)]					
Male	59 (54.6)	78 (54.2)	44 (55)	NS^a^	NS^a^
Female	49 (45.4)	66 (45.8)	36 (45)	NS^a^	NS^a^
Hb (g/dL)	4.3 (3.7-4.7)	6.3 (5–8.02)	9.6 (8.4-10.9)	= .0001	≤ .0001
RBC (10^9^/μL)	1.77 (1.5-2)	2.73 (2.2-3.3)	3.9 (3.6-4.2)	= .0001	≤ .078
WBC (10^6^/μL)	12.1 (8.5-19.1)	11.4 (8.3-15.5)	12.4 (8–15.6)	NS^b^	NS^b^
Lymphocyte(10^6^/μL)	5.3 (2.9-8.1)	3 (1.8-4.8)	1.9 (1.5-3.3)	NS^b^	≤ .0051
Monocytes (10^6^/μL)	0.6 (0.3-1.2)	0.5 (0.2-0.9)	0.7 (0.5-0.9)	NS^b^	NS^b^
Parasitaemia	41322(5060–989003)	52356(5676–1872368)	61000(7396–434024)	NS^b^	NS^b^
HCM (%)	3.3 (0–10)	10 (0–20)	0 (0–3.3)	= .032	≤ .039

Values given are medians numbers (25^th^-75^th^ percentiles) except when indicated otherwise and for parasitaemia [geometric mean (range)]. CM, SMA, UM and AC refer to cerebral malaria, severe malarial anaemia, uncomplicated malaria and asymptomatic controls, respectively. HCM = haemozoin-containing monocytes.

(1) Comparison between SMA and CM.

(2) Comparison between the three groups SMA, CM and UM.

^a^ Statistical significance was obtained using the chi-square test.

^b^ Statistical significance was obtained using the using Mann-Whitney’s test (1) or Kruskal-Wallis’ test (2).

### SMA and CM cases show a similar transient monocyte deactivation phenotype independent of the number of pigmented monocytes

**Figure 1:**
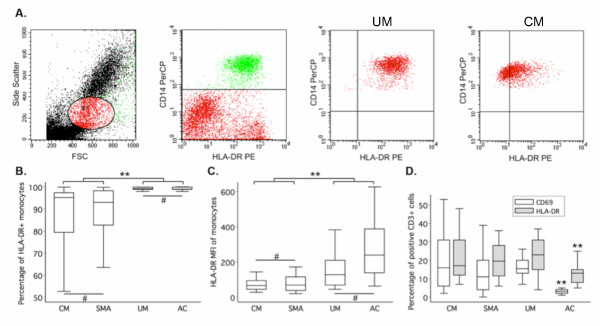
**Monocyte and T cell activation status in the different clinical groups at admission. A.** Monocytes were identified based on their forward (FSC) and side scatter profiles as well as their CD14 positivity (green events). Representative cytograms of monocytes from a UM and a CM patient are shown. **B.** The percentages of CD14^+^ cells (monocytes) positive for HLA-DR were similar between CM and SMA cases and between UM and AC. However, both CM and SMA cases had higher percentages of HLA-DR^+^ monocytes than UM or AC children. **C.** A similar profile was found for the monocyte HLA-DR mean fluorescence intensity (MFI). **D.** The percentages of CD3^+^ cells (T cells) positive for CD69 or HLA-DR, early and late T cell activation markers, respectively were similar between clinical cases but significantly lower in the AC children. Percentages were determined at admission for each group. Data are presented as box plots: the box shows the interquartile range, the line through the box is the median and whiskers indicate the 5^th^ and 95^th^ percentiles. CM, SMA, UM and AC refer to cerebral malaria, severe malarial anemia, uncomplicated malaria and asymptomatic controls, respectively. * denotes *P* ≤ .05; ** denotes *P* ≤ .01; # denotes *P* > .05.

At admission, the percentage of HLA-DR^+^ monocytes and the mean fluorescence intensity (MFI) of HLA-DR expression on monocytes were similar in SMA and CM patients (*P* = .99). Both parameters were higher in UM patients (*P* ≤ .0001) and did not differ from AC children (*P* ≥ .56) (Figure 1A and B). The percentages of HLA-DR^+^ monocytes of SMA (95.9 ± 7.5) and CM (94.6 ± 11.7) patients as well as their HLA-DR MFI (SMA: 155.2 ± 114.4; CM: 147.8 ± 77.4) returned to levels similar to those of AC children 3 days post-admission (*P* ≥ .23).

Since haemozoin phagocytosis was shown to decrease monocyte HLA-DR surface expression [25], the association of monocyte HLA-DR expression with the number of circulating haemozoin-containing (pigmented) monocytes at admission was investigated. In all three clinical groups, the monocyte HLA-DR MFI was unrelated to the percentage of pigmented monocytes (SMA: *P* = .26; CM: *P* = .92; UM: *P* = .69), which did not correlate with parasitaemia (SMA: *P* = .94; CM: *P* = .71; UM: *P* = .72). Of note, the number of pigmented monocytes was associated neither with parasitaemia nor with Hb concentration.

### SMA and CM patients have similar early and late T cell activation markers expression profiles

Because the time between infection and hospital admission - and hence duration of the ongoing infection - may vary across malaria patients, their T cell activation status was assessed using early and late T cell activation markers, namely CD69 and HLA-DR, respectively (Figure1C). SMA and CM patients had comparable percentages of early and late activated T cells (CD69: *P* = .066; HLA-DR: *P* = .87) and similar T cell MFI for these markers were observed in both groups (for CD69: SMA: 26.5 ± 15.6; CM: 21.9 ± 10.4, *P* = .15 ); for HLA-DR: SMA: 64.1 ± 42.1; CM: 42.3 ± 24.6, *P* = .54). The percentages of early and late activated T cells in SMA or CM did not differ from UM patients (CD69: *P* ≥ .22; HLA-DR: *P* ≥ .71). However, malaria patients had higher percentages of early and late activated T cells than AC (CD69, *P* ≤ .0001; HLA-DR, *P* ≤ .0003).

### SMA patients show a lower acute phase IL-10 production compared to CM patients

IL-10 and TNF levels spontaneously released during a 24-hour un-stimulated whole-blood culture, which reflect plasma concentrations at admission [21], showed much lower IL-10 levels in SMA compared to CM (*P* = .003), whereas TNF levels were not different in both clinical groups (*P* = .40) (Table2). Consequently, the TNF/IL-10 ratio at admission was higher in SMA than in CM (median = 8.46 [25^th^ percentile = 2.23; 75^th^ percentile = 23] vs. 2.13 [0.26-14.6], *P* = .015). IL-10 levels in SMA were lower than in UM patients (*P* = .004) and not different from those of AC children (*P* = .45). TNF levels in SMA and CM were comparable to UM (*P* = .40), the lowest levels being in AC children (AC vs. SMA: *P* = .03; vs. CM: *P* = .018; vs. UM: *P* = .003). 

**Table 2:** Spontaneous and stimulated IL-10 and TNF production in malaria patients

		**Spontaneous**	**LPS-stimulated**	**PHA-stimulated**
IL-10	SMA	24.3 (11–39.6)	188.8 (70–342)	55.3 (30.8-176.6)
	CM	201.5 (39–744.8)	623.4 (521.1-1028.25)	608.9 (148.05-725.25)
	UM	100 (22.4-323.2)	198.2 (140.6-349.8)	310 (125.3-494.4)
	AC	12.6 (5.7-17.4)	25.8 (5.1-406)	588.4 (434.2-811)
TNF	SMA	71.1 (24.2-181)	2196 (1595.5-3025.5)	2219 (929.5-3606)
	CM	192.4 (91.5-366.1)	425.6 (217.3-513.7)	876.2 (366.4-1514.5)
	UM	72.6 (42.9-105.2)	1344 (388.2-2461)	1352 (703.7-2534.5)
	AC	55.5 (16.3-94.7)	1265 (544.8-4435)	5857 (2437–9203)

IL-10 and TNF levels were measured by ELISA in the supernatant of un-stimulated whole-blood (spontaneous production) or after LPS or PHA stimulation as described in Methods. Data are presented as median and interquartile range in pg/mL. CM, SMA, UM and AC refer to cerebral malaria, severe malarial anaemia, uncomplicated malaria and asymptomatic controls, respectively.

The spontaneous IL-10 production levels correlated neither with monocyte HLA-DR MFI nor with the percentage of pigmented monocytes in each of the SMA, CM, UM groups or when considering all malaria cases as a single group (*P* ≥ .18).

### SMA patients show a higher IL-10 and TNF monocyte production capacity than CM cases

**Figure 2:**
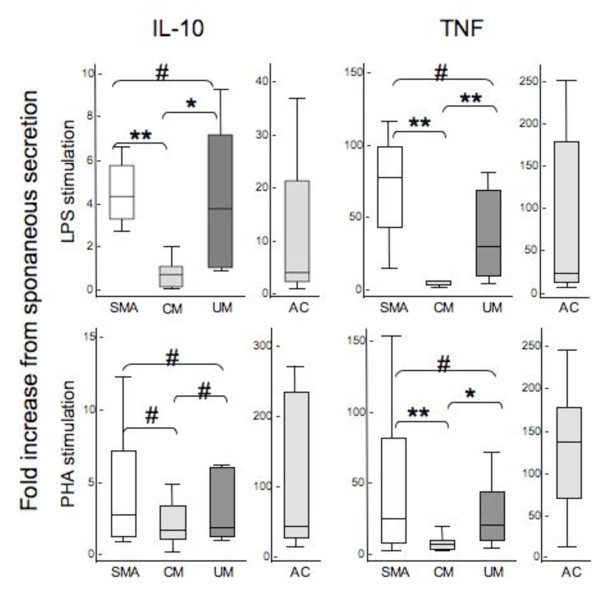
**IL-10 and TNF production capacity in malaria patients.** The IL-10 and TNF production capacity were measured after LPS and PHA stimulation as described in Methods. Fold increases from spontaneous cytokine secretion i.e. un-stimulated samples are presented as box plots: the box shows the interquartile range, the line through the box is the median and whiskers indicate the 5^th^ and 95^th^ percentiles. Statistical significance was determined by Mann–Whitney test. * denotes *P* ≤ .05; ** denotes *P* ≤ .01; # denotes *P* > .05.

In order to determine whether the marked difference in the spontaneous IL-10 production between SMA and CM was linked to a monocyte IL-10 production defect, whole blood LPS stimulation was performed, as it primarily assesses monocyte responses [26]. After 24 hours, IL-10 levels were measured along with TNF levels (Table 2) and fold increases from spontaneous levels calculated (Figure 2). After LPS stimulation, IL-10 and TNF production increased in all groups, including in SMA patients.

Interestingly, the fold (relative) increase in IL-10 production observed in SMA was higher than in CM (*P* = .0082), and not significantly different from the UM (*P* = .09) and AC (*P* = .06) groups (Figure 2). However, the absolute IL-10 levels reached after LPS stimulation in SMA were lower than in CM (*P* = .013) but comparable to UM (*P* = .52) (Table 2). All malaria patients produced higher IL-10 absolute levels than AC (*P* ≤ .0038).

The fold increase in LPS-stimulated TNF production was much higher in SMA than in CM (*P* = .019), UM (*P* = .04) or AC (*P* = .02) (Figure 2) and the absolute TNF levels reached after LPS stimulation were much higher in SMA than in CM (*P* = .0055) and not significantly different from UM (*P* = .26) and AC (*P* = .43) (Table 2).

### Low IL-10 T cell response in SMA

To address the possible involvement of T cells in the specific cytokine imbalance observed in SMA, IL-10 and TNF levels were measured after stimulation with the mitogen PHA, which in these conditions stimulates primarily T cells, with little activation of monocytes [27]. PHA stimulation increased IL-10 and TNF production in all four groups of children (Table 2).

The PHA-stimulated fold-increase in IL-10 production in the SMA group was similar to that of CM (*P* = .18) and UM (*P* = .35), but substantially lower than AC (*P* = .0004) (Figure 2). The absolute IL-10 levels reached after PHA stimulation were lower in SMA than in CM (*P* = .0053), UM (*P* = .036) or AC (*P* = .0007) (Table 2). In contrast, the fold-increase in TNF production was higher in SMA than in CM (*P* = .01), and similar to UM (*P* = .82) (Figure 2). The TNF absolute levels were higher in SMA than in CM (*P* = .0008) or UM (*P* = .036) (Table 2).

## Discussion

The high TNF/IL-10 ratio observed in SMA suggests an imbalanced production of inflammatory cytokines that could contribute to anaemia [5,6]. Whether such an imbalance is an intrinsic characteristic of children with SMA or reflects a specific response pattern to malaria infection involving particular cellular sources has profound implications on the design of intervention strategies to prevent SMA. The data reported here show that SMA patients indeed displayed low spontaneous IL-10 production at admission resulting in higher TNF/IL-10 ratios than CM cases. These findings are consistent with previous studies in Ghana [5,21], but also more recent studies in Southern Zambia [28]. Interestingly, in response to a monocyte or T cell stimulus IL-10 production readily increased in both CM and SMA patients, but SMA patients were characterized by a much higher amplitude of the IL-10 and TNF monocyte response to LPS compared to CM, possibly reflecting different monocyte priming status. However, the absolute levels of IL-10 reached after PHA-stimulation remained modest, much lower than for CM or any other group. This indicates that children experiencing SMA have no inherent incapacity to produce IL-10 and therefore that the imbalanced cytokine response at admission and upon further stimulation *in vitro* likely reflects a specific immunological pattern rather than an intrinsic predisposition to a deficient IL10-production.

The data also provide interesting insights into the immune status of children with CM. Although expression levels of surface activation markers on both lymphocytes and monocytes were similar in CM and SMA, CM patients presented a distinct cytokine expression profile, characterized by spontaneous production of high levels of both TNF and IL-10 but limited increase in TNF production after monocyte or T cell stimulation, suggesting an overall relative low-responsiveness to further stimulation. This points to a distinct functional status of circulating T cells and monocytes in SMA and CM, which both differed from the functional status in UM.

UM children had lower lymphocyte counts, limited monocyte deactivation, balanced IL-10 and TNF levels at admission (both lower than CM, IL-10 higher than SMA) and strong responsiveness to monocyte and T cell stimulation. Thus, based on analysis of circulating cells, the three clinical groups had specific response profiles to the ongoing infection and to further monocyte or T cell stimulation. IL-10 and TNF responses to a T cell stimulus were higher in AC than in any of the three clinical malaria groups, suggesting an impaired T cell responsiveness in malaria (regardless of the clinical presentation) as observed by others [29]. Parasite-related factors may explain the specific IL-10 production profile of SMA patients. Some studies [30,31] but not others [32] have found that haemozoin phagocytosis triggered the production of IL-10 by monocytes and induced a state of monocyte “anergy/reprogramming” associated with a deregulated production of pro-inflammatory cytokines such as TNF [33,34]. However, the lack of association of IL-10 plasma levels with the number of circulating haemozoin-containing monocytes observed here, including in CM patients who have the highest IL-10 levels, does not support a direct impact of haemozoin load on IL-10 production by circulating leukocytes.

There was no significant correlation between the number of pigmented monocytes and haemoglobin. This contrast with results from Casals-Pascual *et al*., although they found a moderately positive correlation (r^2^ = 0,29) [35]. This reflects the unclear relationship between pigmented leukocytes and the disease manifestation or the parasite biomass. The number of circulating pigmented monocytes depends on a complex clearance kinetics [36], which may differ depending on whether anaemia is consecutive to an acute infection or results from a protracted infection.

CD36-dependent adhesion of infected erythrocytes to monocytes may modulate the inflammatory cytokine secretion profile, including IL-10 production [37-39] and the low IL-10 plasma levels in SMA patients may reflect the low CD36-binding capacity of their infected erythrocytes [40]. This is supported by the lower proportion of haemozoin-containing monocytes in SMA relative to CM patients, possibly reflecting the reduced phagocytosis of infected erythrocyte subsequent to CD36 binding [41] but may also merely reflect differences in parasite biomass.

The discrepant IL-10 levels in SMA and CM could result from different types or proportions of IL-10 producing cells. Recent studies suggest that various subsets of CD4^+^ T cells including Tr1 and Th1 are important contributors [42,43]. Compared to CM and UM, SMA cases produced lower absolute levels of IL-10 but higher levels of TNF in response to T cell stimulation. This suggests a T cell functional impairment specific for IL-10 production in children with SMA. Whether this reflects an infection-related cytokine expression programming or an effector/regulatory T cell subset imbalance is unclear. Additional work is needed to elucidate this question, especially since depletion of CD4+ T cells significantly alleviates anaemia in a murine model [44].

Beside T cells, two monocyte subpopulations with different IL-10 producing capacity upon LPS stimulation are now recognized: the regular CD14^bright^CD16^-/dull^ monocytes producing both TNF and IL-10 and the CD14^dim^ CD16^bright^ monocytes producing high levels of TNF and little or no IL-10 [45]. Although the latter subset was recently found to be enriched in SMA children [46], comparable CD14/CD16 cytograms and monocyte CD14 MFI (CM: 234.1 ± 262.3; SMA: 162 ± 95) and CD16 MFI (CM: 10.8 ± 12.2; SMA: 13.9 ± 12.1) were observed here.

A significant but transient down-regulation of HLA-DR expression of circulating monocytes was observed in children with severe malaria, irrespective of the clinical form (SMA or CM). HLA-DR down-regulation has been described for dendritic cells in Kenyan children with acute malaria, but was observed in both mild and severe cases [47]. Phagocytosis of haemozoin and exposure to IL-10 both induce down-regulation of monocyte HLA-DR surface expression [25,48]. However, in the CM and SMA patients studied here, HLA-DR expression was independent from the number of pigmented monocytes and did not correlate with circulating IL-10 levels. The observed HLA-DR down-regulation rather results from the complex integration of multiple anti-inflammatory signals, as observed in severe inflammatory syndromes where it is generally associated with an impaired TNF production capacity in response to further LPS stimulation [49] reflecting a general cellular reprogramming phenomenon of acute inflammatory injuries [50-52]. The impaired TNF production after LPS stimulation observed in CM, but not in SMA, is reminiscent of this cellular reprogramming and suggests interference between the monocyte signalling pathway involved in the overwhelming cytokine production associated with CM and the LPS-triggered MD2 signalling pathway [53,54]. Thus, CM appears as an acute inflammatory syndrome with excessive TNF production by monocytes/macrophages rapidly inducing a high counter-regulatory IL-10 production. In contrast, the high TNF levels observed in SMA would result from a more chronic/sustained production of TNF maintained by an impaired IL-10 regulatory feedback reflecting a specific leukocyte polarization/programming state in SMA.

## Conclusion

The data reported here point towards a specific programming of monocytes and T cells in SMA patients where low IL-10 levels are not due to intrinsic production incapacity but rather to a specific polarization/programming pathway. Differences in the environmental context as well as the intensity and duration of malaria-associated inflammatory stimuli may explain this specific cytokine response. Directions for future work include identification of the parasite factors implicated in the polarization of the immune response and *ex vivo* dynamic functional analysis of specific T cell subsets in children with CM or SMA. Dissecting the infection-acquired changes in cytokine expression profiles associated with SMA and exploring additional upstream and downstream mediators as well as cells possibly implicated in dyserythropoiesis or erythrophagocytosis is of major interest to design intervention strategies.

## Competing interest

The authors have no conflict of interest. Jørgen Kurtzhals has received project funding for unrelated studies from Vifor Pharma, Switzerland and Novo Nordisk, Denmark.

## Authors’ contributions

BG, JK, BDA, LH and CB designed the study. GOA, BG and JK recruited the participants. PB, SL, GAA, JT and MMA generated the data. PB, OMP and CB analysed the data and wrote the manuscript. CB supervised the research and secured the funding. All authors approved the final version of the manuscript.
